# Inequity in health care utilization for common childhood illnesses in India: measurement and decomposition analysis using the India demographic and health survey 2015–16

**DOI:** 10.1186/s12913-021-06887-2

**Published:** 2021-08-27

**Authors:** Tulasi Malini Maharatha, Umakant Dash

**Affiliations:** Department of Humanities and Social Sciences, Indian Institute of Technology Madras, Chennai, Tamil Nadu India

**Keywords:** Common childhood illnesses, Inequality in medical treatment, Erreygers index, Horizontal inequity, Decomposition analysis

## Abstract

**Background:**

Though child mortality has dropped remarkably, it is considerably high in South Asia. Across the globe, 5.2 million children under 5 years of age died in 2019, and India accounts for a significant portion of these deaths. Common childhood illnesses are the leading cause of these deaths. Seeking care from formal providers can reduce these avoidable deaths. Inequity is a crucial blockage in optimum utilization of medical treatment for children. Hence, the present study analyzes the inequalities and horizontal inequities in utilizing the medical treatment for diarrhea, fever, acute respiratory infection (ARI), and any of these common childhood illnesses in India and across the Indian states. The study also attempts to locate significant contributors to these inequalities.

**Methods:**

The study used 0 to 59 months children’s data sourced from the Demographic and Health Survey, India (2015–16). Concentration Index (CI) and Erreygers Corrected Concentration Index (EI) were used to measure the inequalities. The Horizontal Inequity Index (HII) was deployed to estimate inequity. The decomposition method introduced by Erreygers was applied to determine the significant contributors of inequalities.

**Results:**

The EI in medical treatment-seeking for common childhood illnesses was 0.16, while the HII was 0.15. The highest inequality was perceived in the utilization of medical treatment for ARI (0.17). The primary contributing factors of these inequalities were continuum of maternal care (18.7%), media exposure (12%), affordability (9.3%), place of residence (9.1%), mother’s education (8.5%), and state groups (8.8%). The North-Eastern states showed the highest level of inequality across the Indian states.

**Conclusion:**

The study reveals that the horizontal inequity in medical treatment utilization for children in India is pro-rich. The findings of the study suggest that attuning the efforts of existing maternal and child health programs into one seamless chain of care can bring the inequalities down and improve the utilization of child health care services. The spread of health education through different media sources, reaching out to rural and remote places with adequate health personnel, and easing out the financial hardship in accessing medical treatment could be the cornerstone in accelerating the utilization level amongst the impoverished children.

## Background

Investment in child health asserts a greater level of compound benefit, encompassing the lifetime of children, their future generation, and society. Myriads efforts have been undertaken at the global and national levels to improve child health; nonetheless, millions of child deaths occur from preventable causes. Recent estimates elucidate that around 5.2 million under-five years of children deaths occurred in 2019 globally wherein, 47% were contributed by neonatal deaths [1]. Region-wise, nearly 80% of them occurred in Sub-Saharan Africa and South Asia alone. Among countries, India is one of the significant contributors to global child deaths [1]. These deaths were typically caused by major diseases, namely acute respiratory infections (ARI), diarrhea and malaria [2]. Evidence suggests that 1,41,970 under-five children died due to ARI, and 91,270 child deaths were caused by diarrhea [3]. The most tragic fact is that these deaths occurred primarily due to amenable factors such as affordable treatment for childhood illnesses, inadequate nutrition, and access to safe drinking water.

These avoidable causes of child deaths have a strong association with the structural and social determinants of health [4]. Hence, efficacious utilization of child health care services plays a crucial role in reducing these fatalities across different economic strata. Moreover, effective access to child health care services is indispensable so as to attain Sustainable Development Goal (SDG) targets related to neonatal deaths (12 per 1000 live births) and under-five child mortality (25 per 1000 live births) by 2030 [5].

In India, widespread disparity prevails in child health and health care. Few Indian states (Kerala, Tamil Nadu, Maharashtra, Punjab, and Jammu & Kashmir) have achieved the SDG’s target of less than 25 under-five mortality per 1000 live births. At the same time, many states (Assam, Bihar, Chhattisgarh, Odisha, Rajasthan, Uttar Pradesh, and Madhya Pradesh) lag behind the national average of 37 per 1000 live births [6]. Inequitable distribution of child deaths across states or different geographical contours mainly spurred from inequity in the utilization originating from limited access to essential child health care services among impoverished children [7, 8]. While the prevalence of childhood diseases is concentrated among the poor children, the treatment-seeking behaviour is more pronounced amongst the children belonging to the wealthier population [9, 10]. This distorts the fundamental principle of horizontal equity, which states that people in equal need of care should be treated equally [11].

Previous studies examined the association of socio-economic characteristics and utilization of child health care services by adopting a qualitative research approach [12, 13] and quantitative research technique [14, 15]. The existing quantitative studies have employed bivariate and multivariate analysis [16–18]. Few have estimated the coverage gap in the utilization of comprehensive child health care services [19, 20]. These studies highlighted the inequality by estimating the effect of socio-economic factors on the utilization of child health care services. Nevertheless, to the best of our knowledge, none of the studies shed light on inequity in utilization of child health care services. One of the most comprehensive and concise definitions of inequity, rendered by Whitehead (1991), states, “differences in health which are not only unnecessary and avoidable but, in addition, are considered unfair and unjust” [21]. In the present study, inequity exists in the utilization of child health care services if it emerges from the variations in the non-need-based (socio-economic) factors rather than need-based (biological) factors.

Framing or reframing policies to address the problem of persistent inequity in the utilization of child health care services call for robust empirical evidence and operational information on the same. Hence, we attempt to fill this gap by measuring horizontal inequity and inequality in the utilization of child health care services among under-five children who suffered from diarrhea, fever, ARI, and any of these common childhood illnesses in India and across the Indian states. This was measured by employing Erreygers Corrected Concentration Index (EI), an approach which is appropriate when the dependent variable is binary [22]. The study also attempts to unravel the constituents of inequality in utilizing the medical treatment for common childhood illnesses by employing a decomposition analysis.

## Methods

### Data

This study used the data from the India Demographic and Health Survey (2015–16), also known as the National Family Health Survey (NFHS-4), for the analysis. The sample provides comprehensive information on fertility, child mortality, maternal health care and child health, including child nutrition. The NFHS-4 survey sample is representative at national, state, and district levels (for all the 640 districts in India, as of the 2011 Census). It covered 6,01,509 households and 6,99,686 ever-married women (age 15 to 49 years). The collection of data was based on a two-stage stratified cluster sampling. The sampling weights were separately calculated based on the sampling probabilities for each stage. The details of the data collection, sampling technique, survey design, reliability and quality control are described elsewhere [23]. The survey protocols and procedures were reviewed and approved by the IIPS and ICF institutional review board. Hence, the studies using the DHS dataset do not require separate ethical consent. The data is available for public access and we have used the dataset after receiving approval from the DHS program office. The dataset is provided in individual recode files. For the analysis, the individual and the children recode files were merged.

### Conceptual framework

**Fig. 1:**
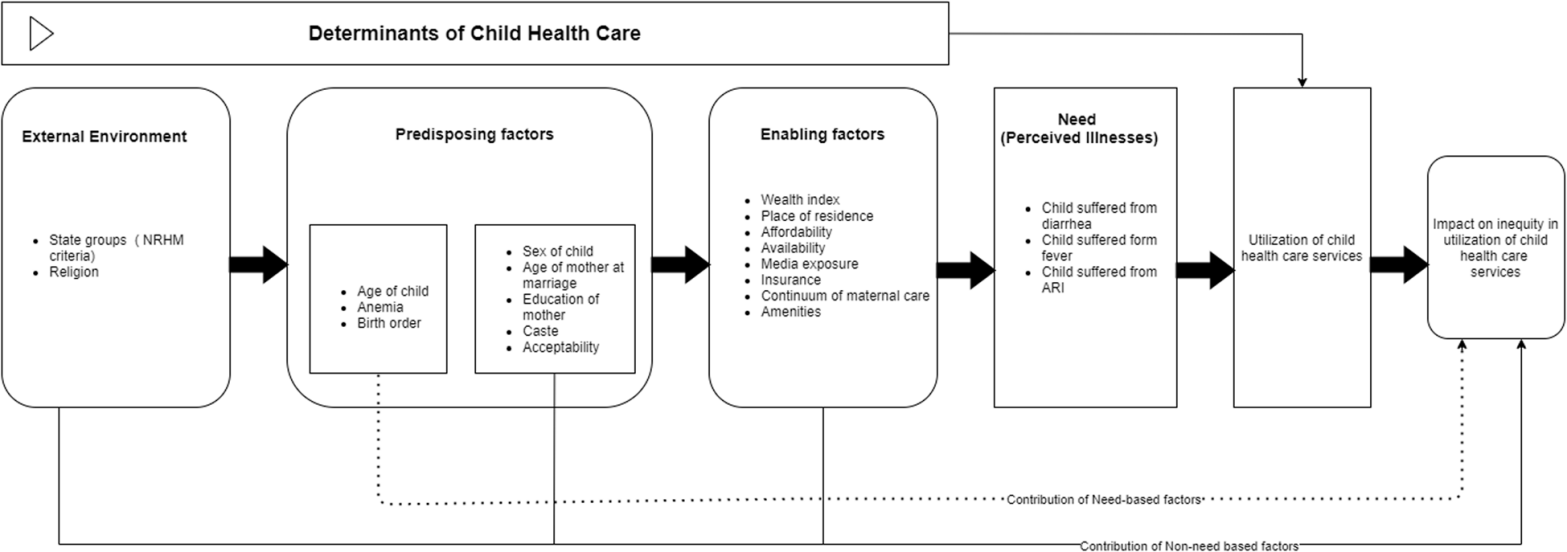
Conceptual Framework of determinants of utilization of health care services for common childhood illnesses. Note: NRHM stands for National Rural Heath Mission

Along the lines of the conceptual frameworks proposed by Anderson [24] and the Commission on Social Determinants of Health [4], this study postulates that both biological needs and social determinants influence child health care utilization. The conceptual framework (Fig. 1) displays the influence of biological and social determinants on the utilization of child health care services. The external environment, such as public policy, social values and other macro variables, mould the predisposing factors. The predisposing factors (including mother and child characteristics and health beliefs, such as attitude towards the health services) are divided into two categories: need-based (legitimate) and non-need-based (illegitimate) factors. The predisposing factors influence the enabling factors, which empower the mother to utilize the health care services for common childhood illnesses. Except for the legitimate predisposing factors, all other determinants are considered as illegitimate factors. The final node of the framework presents the impact of biological and social determinants on the inequity in utilizing child health care services.

### Outcome variables

Children within the age of 0–59 months who suffered from episodes of diarrhea, fever, and ARI or suffered from any one of them are included in this study. The outcome variable represents the utilization of medical treatment services for any of these illnesses. An outcome variable named common childhood illnesses (from now on, CCHI) is created to analyze the overall (diarrhea/fever/ARI) utilization of child health care services. The final sample for the analysis is restricted to the children who suffered from diarrhea or fever or ARI. The services sought from public and private providers are categorized as medical treatment, while treatment sought from informal providers or no care sought are included in the no treatment category. The outcome variable CCHI is coded as 1 (medical treatment) if the respondent sought care for the child from a formal provider; otherwise, it is coded as 0 (no treatment).

### Explanatory variables

The variables used for the horizontal inequity analysis were categorized as legitimate and illegitimate covariates. Mother’s education, continuum of maternal care, mother’s age at marriage, media exposure, wealth index, birth order, age and sex of child, anemia in child, access barriers, insurance, caste, religion, and place of residence were selected from the previous literature pertaining to the utilization of child health care services that were mainly conducted in developing countries [12, 15, 25–28]. In addition, basic amenities and socioeconomic profile (BASEP), and state groups were included to control for the regional variations due to differences in availability of amenities, socio-economic profile, and central government policy in public health.

#### Legitimate factors

The legitimate variables reflect the targeted population’s health status (under-five children in the present study), hence health care needs due to biological factors. Health care need is an elusive concept that has been interpreted in various ways. According to Culyer and Wagstaff, a good definition should include crucial variables such as age, sex and wellness of the population [29]. In this study, age, birth order, and anemia are included as the legitimate variables. Age is a legitimate variable because biological changes occur with a child’s growth [30, 31]. Birth order has also been included as a legitimate factor. The argument behind the inclusion of this variable is that birth order affects the child’s biological standard both directly and indirectly [32, 33]. Due to the lack of nutritional intake by the mother, children of higher birth order are born with lower physical strength. Hence, they need more care than other children [34]. Anemia is also considered as one of the legitimate determinants in the model, because anemic children have a higher risk of falling ill and need more care due to their fragile health status [35, 36].

#### Illegitimate factors

The asset index, also referred to as the ‘wealth index’, is used as a proxy to measure a household’s socio-economic status [37]. The wealth index is a composite measure computed by using the principal component analysis. The selected set of assets of a household and the housing characteristics are included in the index and divided into five (poorest, poor, middle, rich, and richest) quintiles. The details of the wealth index’s construction can be found elsewhere [38]. The wealth index is entered as a rank in the estimation of EI, and its inclusion again as an illegitimate factor could give rise to bias in the decomposition analysis [39]. Thus, the wealth index is not included as an illegitimate factor.

Other illegitimate factors such as the continuum of maternal care (CoC), mother’s education, mother’s age at marriage, sex of the child, caste (schedule caste, schedule tribe, other backward caste, and others), religion (Hindu, Muslim, Christian, other religions), place of residence (rural/urban), media exposure, insurance coverage of the household, state groups, access barriers (availability, affordability, acceptability) and BASEP are included in the analysis. The literature found that female children receive comparatively less attention during illness episodes [40, 41]. Hence, sex of the child is introduced as an illegitimate covariate. The variable CoC is constructed by following the definition of the continuum of maternal care provided by the World Health Organization [42]. It starts with pre-pregnancy care (at least four visits for antenatal care), continues with delivery (delivery by skilled health professional) and ends with post-pregnancy care (postnatal check-up of the mother within 48 h of delivery). The CoC is coded as 1 if a woman avails all the three health cares (complete care), and it is coded as 0 (no care/ inadequate care) otherwise. There are eight questions asked in survey related to accessibility barriers preventing women from accessing medical treatment for themselves. The questions are divided into three categories: availability, affordability and acceptability. The response to ‘getting the money needed for treatment’ is categorized as the affordability barrier. The questions on 1) ‘Distance to health facility’, 2) ‘having to take transportation’, 3) ‘concern that there may not be any health provider’, and 4) ‘concern that there may be no drugs available’ are categorized as the availability barrier. Responses to 1) ‘getting permission to go’, 2) ‘not wanting to go alone’, and 3) ‘concern no female health provider’ are categorized as the acceptability barrier. Though these barriers represent the mother’s health-seeking behaviour for themselves, they are equally relevant in understanding medical treatment utilization among children [15]. Mother’s education is divided into four categories: no education, primary education, secondary education and higher education. The exposure to different media sources (newspaper, radio and television) is categorized into two: no media exposure and access to any media at least once a week.

We have constructed a variable named basic amenities and socioeconomic profile (BASEP) adhering to the categorization provided in the Annual report of the Ministry of Minority Affairs (2012–2013) [43]. The Ministry of Minority Affairs of India has identified the districts with 20% or more minority population and then categorized them by backwardness parameters (basic amenities and socio-economic profile). The indicators considered for basic amenities are percentage of household with pucca wall, safe drinking water, electricity, and water closet latrines; while literacy rate, female literacy rate, work participation rate, and female work participation are included to present the socioeconomic profile of the districts. Inclusion of BASEP as a covariate in the analysis could capture the inequality in seeking care for children due to district level variation in ethnicity, availability of amenities and socioeconomic profile. The BASEP is divided into three categories - A, B and C. The districts below the national average in both socio-economic and basic amenities parameters come under Category A. Category B comprises the districts that fall below the national average either in the socio-economic or basic amenities parameter. Category C contains the remaining districts that do not fall under any of the above categories (A/B). They are also not listed in the report.

A variable state group is generated adhering to the National Rural Health Mission (NRHM)^1^ criterion for high focus states and non-high focus states (states with 25% or less institutional delivery were termed as high focus states and others as non-high focus states). Though North-Eastern states are grouped under high focus states, they differ in geographical location and budgetary allocation. All the North-Eastern states are covered mainly by mountainous terrain, and several barriers exist in transportation and communication. In addition, they are categorized under “Special Category States” by the government of India; hence, they have a separate provision for financial transfer from the Central government [44]. Therefore, high focus states have been divided into high focus states and high focus North-East states [list of the states under each category are given in Appendix 1]. High focus states receive relatively more funds through NRHM from the central government to develop health infrastructure and extend the coverage of maternal and child health care services. Thus state group is included in the analysis to encapsulate the effect of central health policy to promote access to essential health care services.

### Analytical methods

The concentration curve (CC) has been used to provide a visual presentation of the distribution of the utilization of health care services across the socio-economic status (SES). The CC plots the cumulative percentage of the utilization variable against the cumulative percentage of the population, ranked by wealth quintiles (a proxy for SES), starting from the poorest and ending with the richest quintile. The inequality across the wealth quintiles in utilizing child health care services is quantified by deploying two rank dependent indicators - Concentration Index (CI) and Erreygers Corrected Concentration Index (EI). The CI is defined as twice the area between the CC and the line of equality (45- degree line) [45]. If the CC coincides with the line of equality, the value of CI is 0 implying no inequality. The CI ranges between − 1 and + 1. A positive value of the CI indicates a pro-rich inequality.

Indirect standardization is adapted for standardization of distribution [45]. This method facilitates the prediction of a distribution from the actual distribution of an outcome variable across SES. In this method, the association between the legitimate variables and the outcome variable is estimated conditional on the illegitimate variables. The distribution of the indirectly standardized outcome variable can be inferred as the distribution of a health outcome that would be observed irrespective of the distribution of illegitimate factors across wealth quintiles.

The EI standardizes the uncorrected CI by adjusting the CI for the bounded nature of the outcome variable. In the context of the binary outcome variable, the EI is the CI multiplied by four times the outcome of interest’s mean [22]. The EI satisfies the four most desirable properties (transfer, mirror, level dependence and cardinal invariance) of the rank dependent indices [46].

The income-related distribution of actual utilization describes inequality, while the need-standardized utilization describes inequity. The Horizontal Inequity Index (HII) is defined as the differential utilization across different levels of need. The HII can be obtained by subtracting the contributions of all legitimate variables from the unstandardized EI. Following the method proposed by Doorslaer et al. [47], the present study estimates the HII by subtracting the legitimate factors’ contribution from the unstandardized EI. Unstandardized EI refers to the value of the EI calculated with the actual distribution of the utilization variable across the wealth index provided by the data.

The decomposition analysis unravels the components of the measured inequality (both legitimate and illegitimate). The study adheres to the decomposition method proposed by Erreygers [22]. The decomposition analysis results presented with coefficient values of covariates, EI values of the covariates, and absolute and relative contribution to the inequality. Absolute inequality is a product of the coefficient and EI values of the covariates. A positive or negative value in the absolute contribution of the explanatory variable means that the inequality in a dependent variable would decrease if the variable distributes equally across the wealth quintiles. The relative contribution, which is a ratio of the absolute contribution to the EI, represents the percentage contribution.

The analysis has been conducted using STATA version 15. CI and EI are calculated using the ‘conindex’ command proposed by O’Donnell et al. [48]. The bivariate analysis is performed for each control variable with the outcome variables. Only the covariates having *p*-values less than 5% level of significance are included in the model.

## Results

**Fig. 2:**
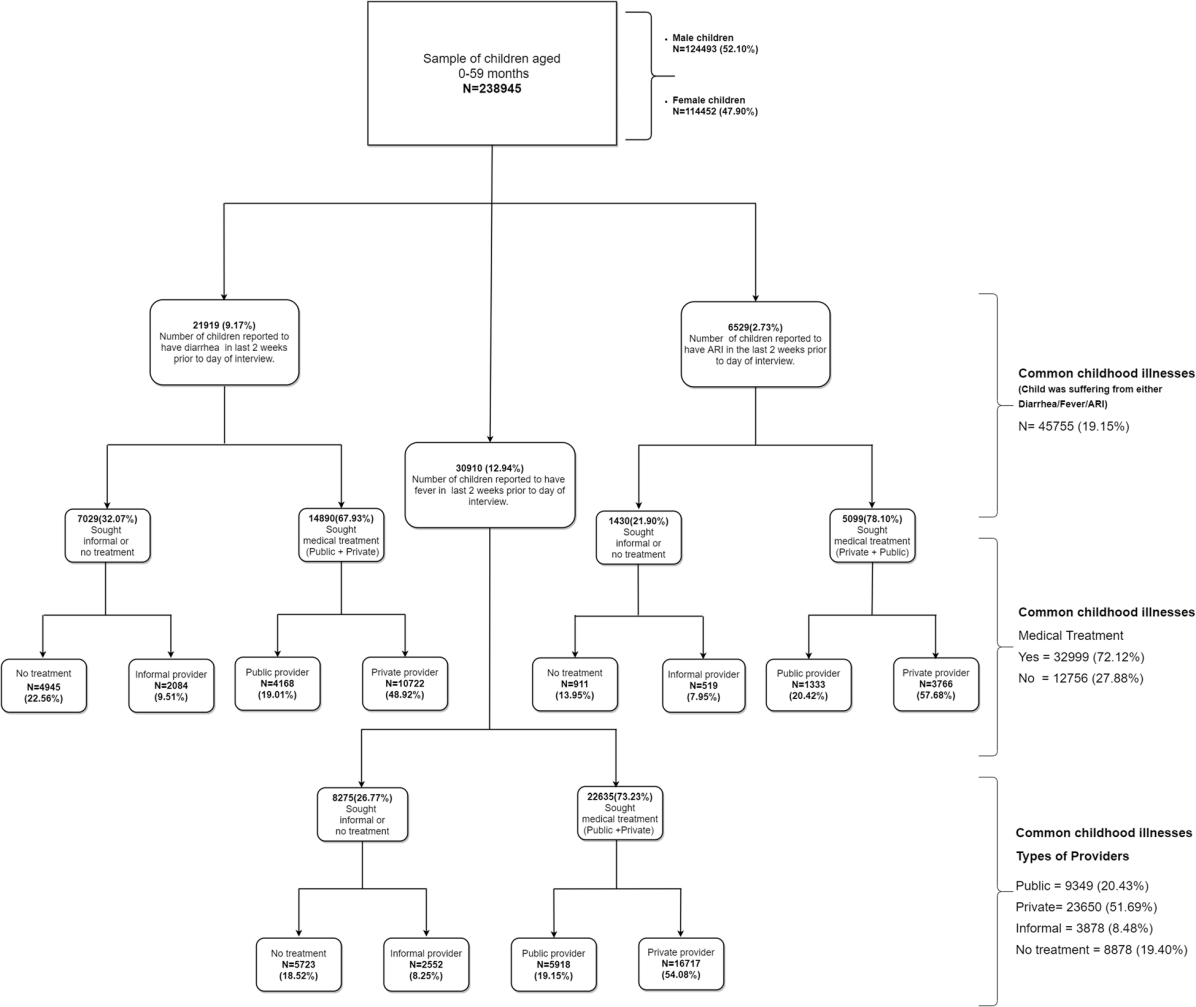
Flow chart of child’s illnesses and health services utilization reported by mother

Figure 2 demonstrates the prevalence of common childhood illnesses and the percentage of sought treatment from different providers. The prevalence rate of diarrhea, fever, ARI and CCHI were 9.17, 12.94, 2.73 and 19.15%, respectively. Considering individual illnesses, fever had the highest prevalence, followed by diarrhea and ARI. The percentage of children suffering from ARI sought the highest medical treatment (78.10%). In case of the types of providers, private providers dominated over public providers in the utilization of health care services, both for individual diseases (Private providers for diarrhea treatment = 48.92%, fever treatment = 54.08%, ARI treatment = 57.68%) and the CCHI (private provider =51.69%). However, there were many children who did not receive any treatment (Diarrhea =22.56%, Fever = 18.52%, ARI = 13.95%, and CCHI = 19.40%) or received informal treatment (diarrhea = 9.51%, fever =8.25%, ARI = 7.95%, and CCHI = 8.48%).

**Fig. 3:**
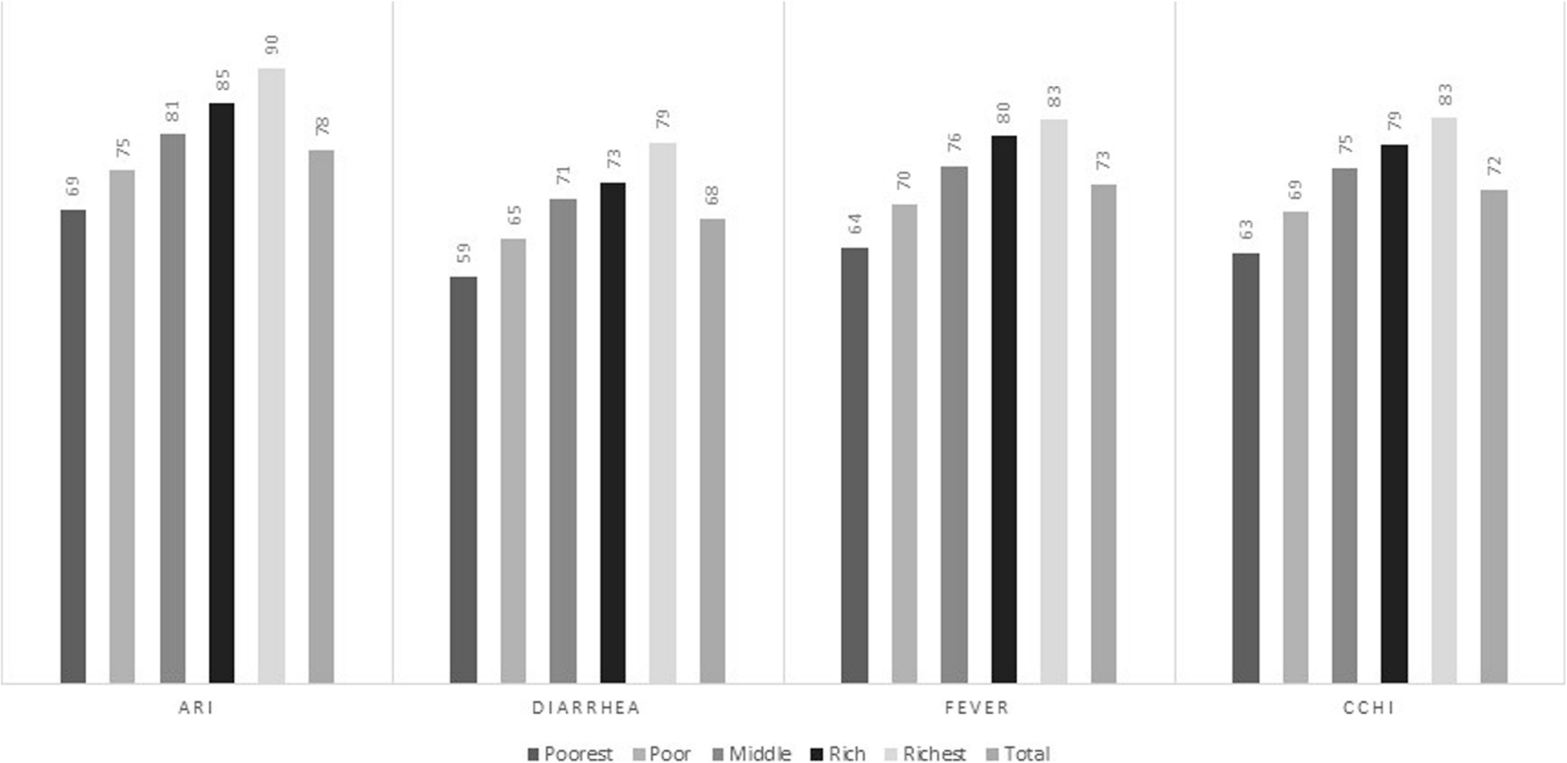
Distribution of wealth index with corresponding medical care utilization for common childhood illness

Figure 3 represents the distribution of medical treatment utilization in percentage for children who suffered from diarrhea/fever/ARI/CCHI across wealth quintiles. The utilization of medical treatment is 72% for CCHI. It could be observed from Fig. 3 that the rate of utilization improved as the wealth quintile proceeds from the poorest to the richest quintile. A pro-rich [Richest (Q5) – Poorest (Q1) > 0] distribution could be perceived, with an absolute gap of 20% (Q5-Q1) for the CCHI. Similarly, pro-rich inequality was observed in medical treatment utilization for diarrhea, fever, and ARI, with absolute differences of 21%, 20%, and 19%, respectively.

Table 1 provides the descriptive statistics of the sample considered for analysis in this study. The table shows that utilization of health care services had decreased with the child’s growth (76% for 0–1 and 69% for 4–5 years old children). Mothers with the complete CoC utilized 13% more medical treatment for their children than their counterparts. Children residing in urban areas sought 8% more medical treatment than children from rural areas. Non-high focus states showed a higher utilization of child health care services. Women who faced any access barrier in utilizing health care services for their health sought less health care services (around 10%) for their children. Mothers with higher education sought 15% more health care for their children in contrast to mothers with no education. Exposure to media also helped to catalyze the utilization of medical treatment as 11% more utilization was reported by the mothers who accessed media at least once a week.

**Table 1:** Descriptive statistics of medical treatment for CCHI according to background characteristics

Variable	Total	Proportion (95%CI)	SE	Medical Treatment (%)
***Wealth index***				
Poorest	9691	0.26 (0.254–0.263)	0.002	6096(62.91)
Poor	8503	0.23 (0.223–0.231)	0.002	5863(68.95)
Middle	7670	0.20 (0.201–0.209)	0.002	5784(75.41)
Rich	6821	0.18 (0.178–0.186)	0.002	5394(79.08)
Richest	4764	0.13 (0.124–0.131)	0.002	3947(82.85)
**Child’s age**				
0–1 year	5883	0.16 (0.153–0.161)	0.002	4470(75.98)
1–2 years	11,185	0.30 (0.294–0.303)	0.002	8200(73.31)
2–3 years	8079	0.22 (0.212–0.220)	0.002	5825(72.10)
3–4 years	6794	0.18 (0.178–0.185)	0.002	4772(70.23)
4–5 years	5508	0.15 (0.144–0.151)	0.002	3818(69.31)
**Birth order**				
> 2	11,900	0.32 (0.313–0.322)	0.002	7999(67.22)
< =2	25,549	0.68 (0.678–0.687)	0.002	19,085(74.70)
**Anemia**				
Not anemic	14,216	0.38 (0.375–0.385)	0.003	10,281(72.32)
Anemic	23,233	0.62 (0.615–0.625)	0.003	16,803(72.31)
**Sex of child**				
Female	17,137	0.46 (0.453–0.463)	0.003	12,168(71.00)
Male	20,312	0.54 (0.537–0.547)	0.003	14,917(73.44)
**Continuum of maternal care**				
No care/incomplete care	23,151	0.62 (0.613–0.623)	0.003	15,615(67.45)
Complete care	14,298	0.38 (0.377–0.387)	0.003	11,469(80.22)
**Religion**				
Hindu	29,144	0.78 (0.774–0.782)	0.002	21,094(72.38)
Muslim	6699	0.18 (0.175–0.183)	0.002	4718(70.44)
Christian	670	0.02 (0.017–0.019)	0.001	503(75.06)
Others	936	0.02 (0.023–0.027)	0.001	769(82.18)
**Caste**				
Schedule Caste (SC)	8188	0.22 (0.214–0.223)	0.002	5936(72.50)
Schedule Tribe (ST)	3399	0.09 (0.088–0.094)	0.001	2293(67.46)
Other Backward Caste (OBC)	17,124	0.46 (0.452–0.462)	0.003	12,431(72.60)
Other	8738	0.23 (0.229–0.238)	0.002	6424(73.52)
**Residence**				
Rural	27,968	0.75 (0.742–0.751)	0.002	19,616(70.14)
Urban	9481	0.25 (0.249–0.258)	0.002	7469(78.77)
**Media exposure**				
No media	13,492	0.36 (0.355–0.365)	0.002	8860(65.67)
At least one media	23,957	0.64 (0.635–0.645)	0.002	18,225(76.07)
**Mother’s education**				
No education	10,895	0.29 (0.286–0.296)	0.002	7142(65.55)
Primary education	5755	0.15 (0.150–0.157)	0.002	4074(70.79)
Secondary education	17,138	0.46 (0.453–0.463)	0.003	12,904(75.29)
Higher education	3661	0.10 (0.095–0.101)	0.002	2965(80.99)
**Age at marriage**				
< 18	14,604	0.39 (0.385–0.395)	0.003	10,029(68.67)
> =18	22,845	0.61 (0.605–0.615)	0.003	17,056(74.66)
**Insurance**				
Yes	32,180	0.86 (0.856–0.863)	0.002	23,071(71.70)
No	5269	0.14 (0.137–0.144)	0.002	4013(76.16)
**Availability**				
No problem	4615	0.12 (0.120–0.127)	0.002	3563(77.20)
Not a big problem	20,759	0.55 (0.549–0.559)	0.003	15,108(72.78)
Big problem	12,075	0.32 (0.318–0.327)	0.002	8414(69.70)
**Affordability**				
No problem	15,832	0.42 (0.418–0.428)	0.003	12,053(76.13)
Not a big problem	10,663	0.28 (0.280–0.289)	0.002	7692(72.13)
Big problem	10,953	0.29 (0.288–0.297)	0.002	7340(67.02)
**Acceptability**				
No problem	6764	0.18 (0.177–0.185)	0.002	5169(76.43)
Not a big problem	20,341	0.54 (0.538–0.548)	0.003	14,686(72.20)
Big problem	10,344	0.28 (0.272–0.281)	0.002	7229(69.88)
**Basic Amenities and Socio-Economic Profile (BASEP)**				
Category A	5684	0.15 (0.148–0.155)	0.002	3725(65.54)
Category B	2354	0.06 (0.060–0.065)	0.001	1782 (75.68)
Category C	29,411	0.79 (0.781–0.789)	0.002	21,577(73.37)
**State groups**				
Non-high Focus state	13,660	0.36 (0.360–0.370)	0.002	10,732(78.57)
High Focus North-East state	887	0.02 (0.022–0.025)	0.001	463(52.16)
High Focus state	22,902	0.61 (0.607–0.616)	0.003	15,889(69.38)

*CI* Confidence Interval, *SE* Standard Error

**Fig. 4:**
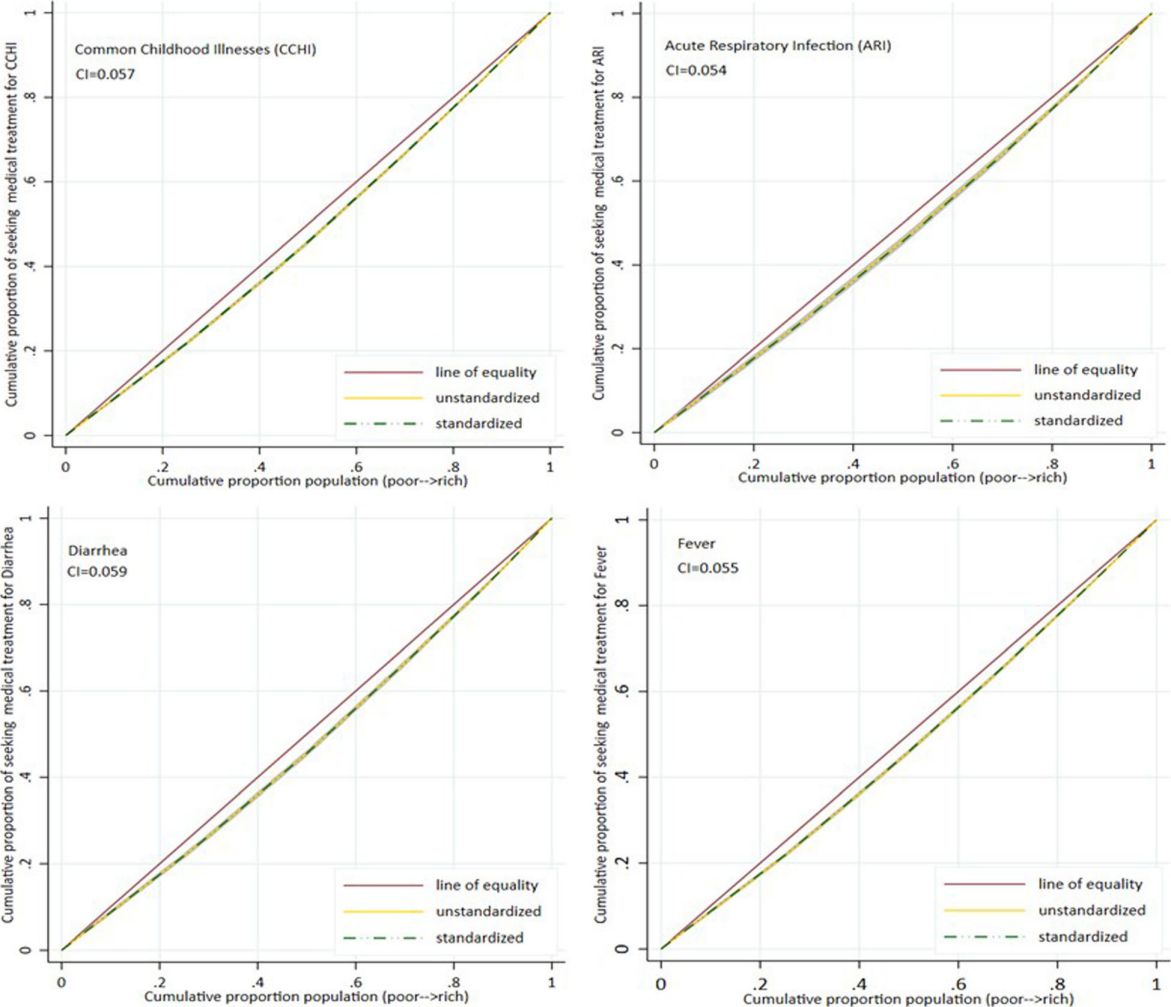
Standardized and unstandardized concentration curves for utilization of medical treatment for Fever,Diarrhea, ARI, and CCHI

Figure 4 shows the standardized and unstandardized CCs for diarrhea, ARI, fever and CCHI. The standardized and unstandardized CIs are quite close to each other due to the least contribution of legitimate factors to the inequity in utilizing health care services for children. Therefore, the standardized and unstandardized CC coincide. We have reported only standardized CIs as unstandardized CIs had similar values.

Table 2 presents the CI, EI, and HII values for the utilization of health care services for diarrhea, fever, ARI and CCHI. While estimating the HII for individual illnesses (diarrhea, fever, ARI), the alternative two illnesses are entered as legitimate covariates in the model. The CI was highest for diarrhea (CI = 0.059), while the EI was highest for ARI (EI =0.163). Inconsistencies in the index values of the CI and EI across illnesses might be due to the bounded nature of the health variable, where the standard CI is not an appropriate measure of the inequality. However, inequality in the utilization of health services for diarrhea and fever has approximate values in both indices. ARI also had the highest HII value, indicating a relatively higher contribution (0.173) of illegitimate factors to the utilization of health care services compared to the other two illnesses. The contribution of legitimate factors to total inequality is minimal. However, if the need is the only factor to decide utilization, it would have been pro-poor for diarrhea (− 0.006) and ARI (− 0.01).

**Table 2:** CI, EI and HI of Childhood Illnesses

	Fever	Diarrhea	ARI	CCHI
**Concentration Index (CI)**	0.055	0.059	0.054	0.057
**Erreygers Index (EI)**	0.153	0.149	0.163	0.155
**Legitimate**	0.001	−0.006	−0.010	0.005
**Illegitimate**	0.116	0.116	0.144	0.115
**Residual**	0.035	0.039	0.029	0.035
**Horizontal Inequity Index (HII)**	0.152	0.154	0.173	0.150

*ARI* Acute Respiratory Infection, *CCHI* Common Childhood Illnesses

**Fig. 5:**
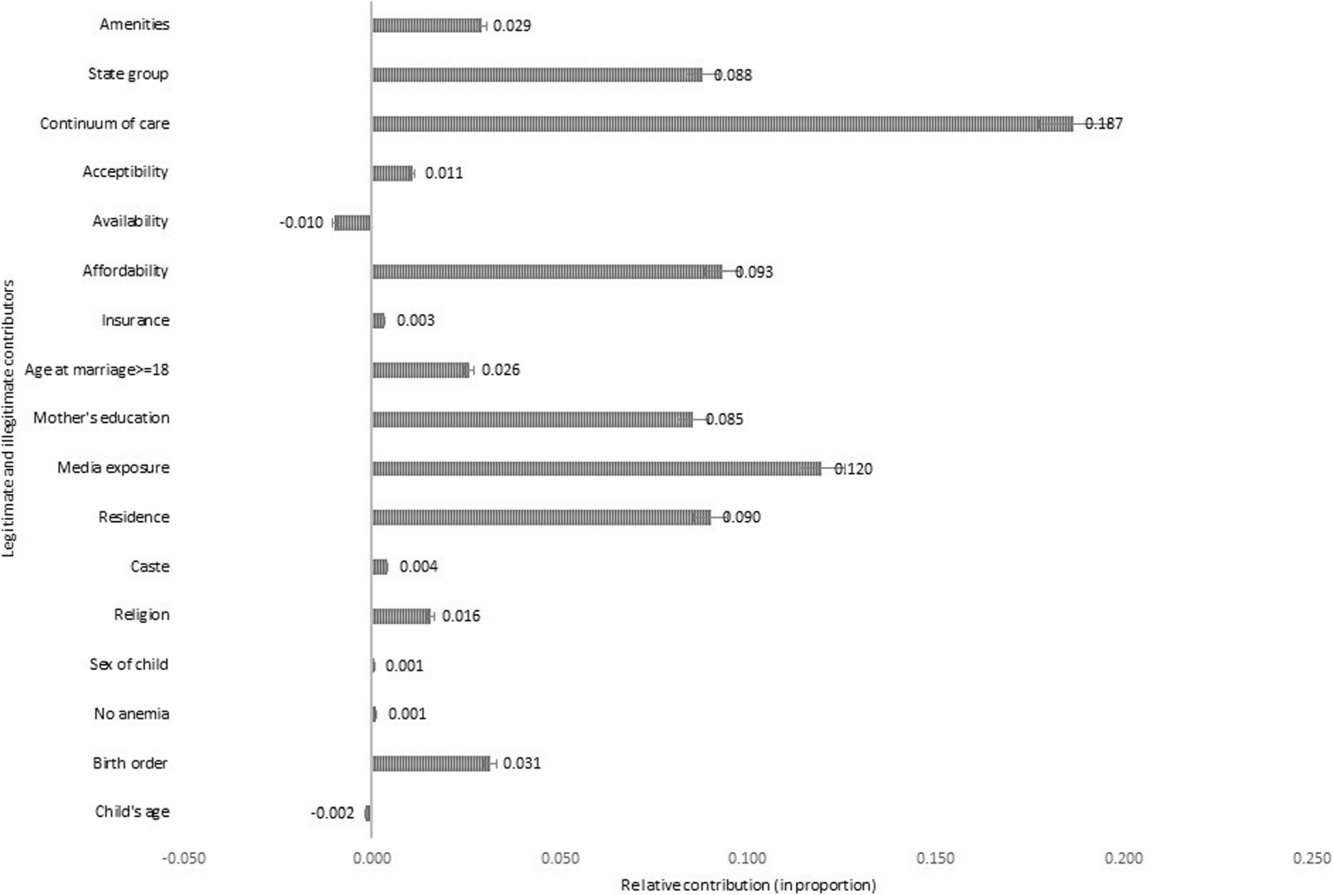
Decomposition of the Erreygers Corrected Concentration Index: The relative contribution. Note: Error bars denote the 95% confidence intervals. CCHI: Common Childhood Illnesses

Figure 5 presents the decomposition results of the EI and reported the relative contribution of contributors to EI. The decomposition analysis was conducted for the CCHI only. The level of inequalities in the utilization of medical treatment for all three diseases was not dispersed widely. Hence, it could be represented through the CCHI (central point to/average of all the three illnesses). The illegitimate factors contributed substantially (74.8%) to total inequality, while legitimate factors contributed only 3% (birth order). The crucial illegitimate contributors to the EI were the CoC (18.7%), media exposure (12%), residence (9.2%), affordability barrier (9.3%), mother’s education (8.5%), and state groups (8.8%). Other significant illegitimate variables that contributed less than 3% to the EI were BASEP (2.9%), age at marriage (2.6%), and religion (1.6%).

Table 3 provides information on the coefficients, EI values, absolute and relative contribution of the covariates. The association between the covariates and the health care utilization variable can be interpreted from the coefficient values. The EI value of the covariates represented the socio-economic status-related inequality in the factor. A positive value of the factor’s EI indicates that the variable has pro-rich inequality induced by the wealth index, while a negative value ascertains that the inequality in the distribution of the covariate is not due to the wealth index.

**Table 3:** Detailed decomposition analysis of the EI estimated for CCHI

Variables	Coefficient	EI	Absolute	Relative
**Need based**				
*Child’s age (base category: Year 0)*			0.000	− 0.003
Year 1	0.003	−0.007	0.000	0.000
Year 2	−0.009	−0.007	0.000	0.000
Year 3	−0.027^a^	−0.001	0.000	0.000
Year 4	−0.036^a^	0.009	0.000	−0.003
*Birth order (base category: > 2)*				
Birth order <= 2	0.018^a^	0.266	0.005	0.031
*Anemia*				
Not anemic	0.003	0.055	0.000	0.002
**Non-need based**				
*Sex of child (base category: Female)*				
Male	0.024^a^	0.004	0.000	0.002
*Continuum of maternal care (base category: No care/Incomplete care)*				
Complete care	0.076^a^	0.380	0.029	0.187
*Religion (base category: Muslim)*			0.002	0.016
Hindu	−0.011	−0.056	0.001	0.004
Christian	0.029	0.014	0.000	0.003
Other religion	0.046	0.030	0.001	0.009
*Caste (base category: Schedule Caste (SC))*			0.001	0.004
Schedule Tribe (ST)	−0.030^a^	−0.110	0.003	0.021
Other Backward Caste (OBC)	−0.007	0.038	0.000	−0.002
Other caste	−0.014	0.178	−0.002	−0.016
*Place of delivery (base category: Rural)*				
Urban	0.031^a^	0.454	0.014	0.091
*Media exposure (base category: No media)*				
At least one media	0.031^a^	0.602	0.019	0.120
*Mother’s education (base category: No education)*			0.013	0.085
Primary education	0.019^b^	−0.103	−0.002	−0.012
Secondary education	0.020^a^	0.313	0.006	0.040
Higher education	0.040^a^	0.225	0.009	0.058
*Age at marriage (base category: < 18 years)*				
> = 18 years	0.015^b^	0.264	0.004	0.026
*Insurance (base category: No)*				
Yes	0.016^c^	0.032	0.001	0.004
*State groups (base category: North East high focus states)*			0.014	0.088
Non-high focus states	0.197^a^	0.353	0.070	0.450
High focus states	0.164^a^	−0.341	−0.056	−0.362
*Affordability (base category: Big problem)*			0.014	0.093
Not a big problem	0.030^a^	−0.020	− 0.001	−0.004
No problem	0.052^a^	0.290	0.015	0.097
*Availability (base category: Big problem)*			−0.002	−0.010
Not a big problem	0.001	0.003	0.000	0.000
No problem	−0.009	0.168	−0.002	− 0.010
*Acceptability (base category: Big problem)*			0.002	0.011
Not a big problem	0.008	−0.057	0.000	−0.003
No problem	0.012	0.174	0.002	0.014
*Basic Amenities and Socio-economic Profile (BASEP) (base category: A)*			0.005	0.029
Category B	0.044^a^	0.039	0.002	0.011
Category C	0.022^b^	0.126	0.003	0.018
*Residual*			0.035	0.222

^a^1% level of significance, ^b^5% level of significance, ^c^10% level of significance; *EI*: Erreygers Index

From the coefficient values, it could be inferred that the mother’s education and CCHI were positively related (primary education = 0.02, secondary education = 0.02, and higher education = 0.04). The coefficients for the age of children showed a negative value for the 2 to 4 years category, which means that the probability of utilization of medical treatment decreased with an increase in age. The residual term indicates the unobserved heterogeneity that affects the observed socio-economic inequality in the dependent variable (0.035).

The detailed decomposition analysis demonstrates how the different categories within the covariates contribute individually to the EI and are associated with the health variable and the wealth index. The relative contribution of the state group to the EI was 8.8%. Within the state groups, the High-focus states (− 36.2%) showed a negative value while the Non-high-focus states (45%) represented pro-rich inequality over the base category of the North-Eastern states. It could be discerned that the inequality in the Non-high focus states mostly attributed to wealth, while children from the High focus states might be facing inequality due to other social determinants as well. Similarly, the mother’s primary education (− 1.2%) depicted a pro-poor distribution while her secondary (4%) and higher education (5.8%) displayed a pro-rich distribution. The BASEP contributed around 3% to the EI. Group B districts contributed less to the inequality (1.1%) as compared to the group C districts (1.8%). Among the three access barriers, affordability was seen as one of the major contributors to the EI. The distribution of seeking medical care for those who did not face this barrier was pro-poor (− 0.4%).

### State level analysis

**Fig. 6:**
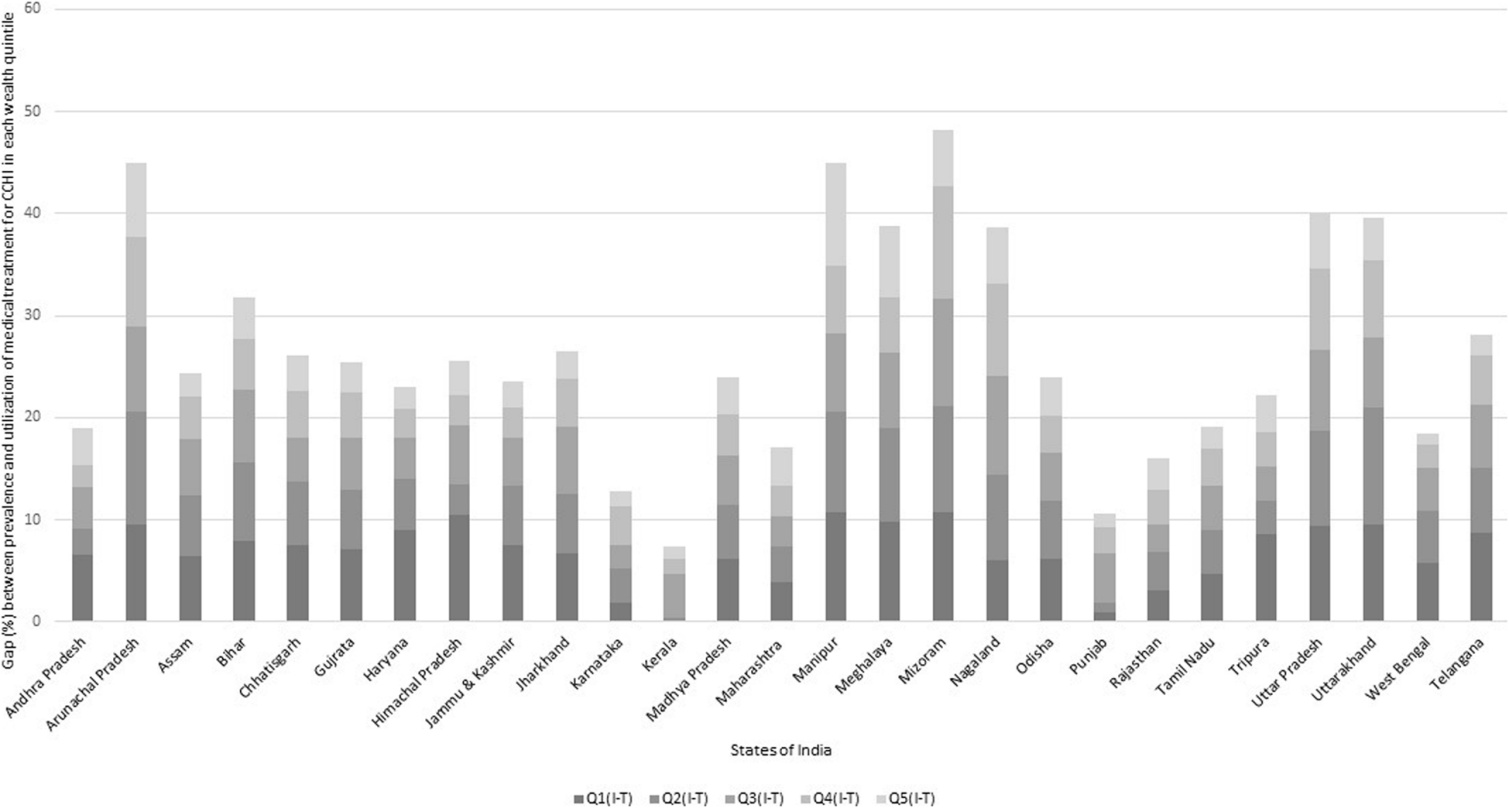
Gap between the prevalence and utilization of medical treatment for CCHI across wealth quintiles in the states of India. Note: i. Q1 (poorest), Q2 (Poor), Q3 (Middle), Q4 (Rich), Q5 (Richest) are the wealth quintiles. ii. I (Illnesses) – T (Medical Treatment) is in percentage. iii. CCHI: Common Childhood Illnesses

Figure 6 illustrates the gap (in percentage) between prevalence and utilization of medical treatment for CCHI in each wealth quintile across the states of India. All the Union Territories (UTs), Sikkim, and Goa are dropped from the state-level analysis due to a limited number of observations. The stacked column is divided into five parts, representing the interspace of prevalence and utilization in each quintile. Across the states, the lowest gap is encountered by Kerala, followed by Punjab, Karnataka, Rajasthan, and Maharashtra. Mizoram witnessed the highest level of total gap. Other North-Eastern states (Arunachal Pradesh, Manipur, Meghalaya and Nagaland) also reported a prominent gap. Kerala and Punjab exhibited less than 1% gap in the poorest quintile while Manipur, Mizoram, and Himachal Pradesh showed more than 10% gap. Manipur, Meghalaya, and Arunachal Pradesh also had a higher gap in their richest quintiles as compared to the other states.

**Fig. 7:**
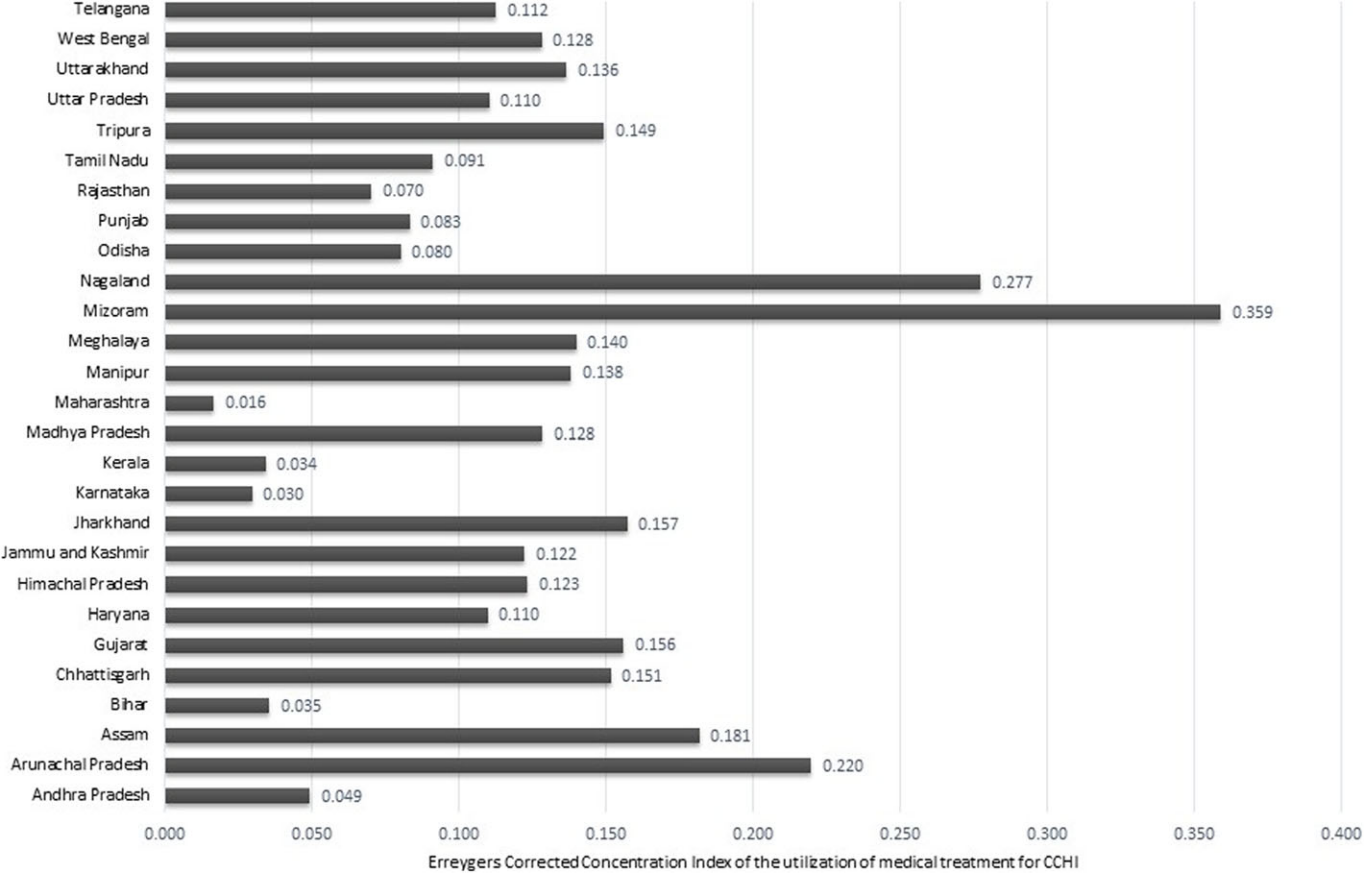
Erreygers Corrected Concentrated Index values for the utilization of medical treatment for CCHI across the Indian States. Note: CCHI: Common Childhood Illnesses

Figure 7 elucidates the EI values of the states. The highest level of inequality was reported in Mizoram (0.36), followed by Nagaland (0.28), Arunachal Pradesh (0.22), and Assam (0.18). The lowest value of EI was found in Maharashtra (0.02), while Karnataka and Kerala hover around the same level of pro-rich inequality (0.03). Surprisingly, Bihar showed a lower value of EI (0.04) even though the child and maternal health indicators of this state are not very encouraging. All the North-Eastern states (Tripura, Nagaland, Mizoram, Meghalaya, Manipur, Arunachal Pradesh and Assam) had EI values that approximate 0.14 and above, while the southern states, except for Tamil Nadu (0.09), had an EI of less than 0.05.

## Discussion

In this paper, we have measured the inequity and inequality in the utilization of medical treatment for diarrhea, fever, ARI, and CCHI. Our results ascertained that the distribution of inequality was pro-rich. The HIIs showed that the illegitimate factors were significant contributors to the total inequity. It implies that inequity prevails in the utilization of medical treatment for under-five children due to the unequal distribution of socio-economic determinants in India. Time and again, researchers have asserted that the household’s economic status positively impacts the utilization of child health care services [25, 27, 28]. Poor households might lack the financial capacity to pay for their children’s medical treatment and resorted to home or non-institutional treatment.

The CoC had significantly determined the utilization of child health care services with the highest contribution (18.7%) to pro-rich inequality. Many studies found that the CoC was mostly concentrated among the wealthy population [49, 50]. The contribution was higher because the linkage between maternal and child health care is decisive. Mothers who availed the CoC during their pregnancy life cycle might have been more likely to seek necessary health care services for their children [51]. The utilization of formal health care for their health could also be a driving factor for preferring care from a formal provider for their children over informal or no care [26, 52].

In line with the previous literature, this study found that the mother’s education played a prominent role in treatment-seeking for children and contributed to pro-rich inequality (8.5%) [27, 28, 53]. Women belonging to the rich quintile always have a better opportunity for education. Besides, their decision-making power within the household is relatively better compared to a woman with no education. Also, a mother with higher education has a greater awareness of childhood illnesses and the availability of health care facilities [28].

The lack of information regarding the availability of health care services, public health policies, and the necessity of utilizing health care at the right time and from the right place are severe impediments to medical care utilization for children. The dissemination of essential information through media can work as an essential tool to improve child health care utilization. The mothers exposed to any form of media understand the danger signs/ symptoms of childhood diseases better and make a conscious effort for timely treatment [54]. Previous studies had also found a positive impact of media exposure on the utilization of maternal and child health care services [25, 53, 55]. Since access to the various media sources (newspaper, radio and television) is more convenient and affordable for the wealthy population, its concentration was higher among them.

Analysis of the barriers to access health care services demonstrated the affordability issue (9.3%) to be a major impediment. Several studies in India concluded that health expenditure is catastrophic for the poor section [7, 56]. A study conducted in Lucknow, Uttar Pradesh, found that families belonging to the lower-income group were reluctant to seek health care services for their children [17].

The present study reveals that the place of residence played a crucial role in the unequal distribution of treatment-seeking behaviour for child health care (9.2%). It is corroborated by the findings of previous studies [25, 27]. It could be possibly attributed to the poor infrastructure in rural areas. Health facilities in rural areas often grapple with the lack of proper infrastructure, unavailability of drugs and equipment and under-staffing. This ultimately leaves rural residents with no option but to seek care from easily accessible and available informal providers (traditional healers, medicine shops, quacks) [16, 55, 57].

We found that, overall, the state groups have a pro-rich contribution. However, the high-focus group showed negative while the non-high-focus group states reported a positive sign in their relative contribution towards total inequality. This indicates that, in the high-focus states, the determinants apart from the wealth index had a significant contribution towards inequality in seeking care for children. The NHM has made some special provisions and allocated an additional budget for the high-focus states to upgrade infrastructure and amplify the health workforce to meet the increasing needs of the people. These initiatives possibly explain why high-focus group states were not significantly affected by the inequality in medical treatment utilization caused by wealth differentials. This argument was supported by a previous study, which revealed that the socio-economic inequalities in high-focus states declined after the implementation of NRHM [58].

A state-level inequality analysis was conducted to portray the dispersed distribution of inequality estimates across the Indian states. The inter-state comparison enables us to comprehend the regional disparity in medical treatment utilization among under-five children in India. Maharashtra demonstrated the prevalence of the lowest pro-rich inequality (EI = 0.016) compared to other Indian states. A situational analysis conducted in Mumbai delineates that though development in maternal health is elusive, the city has achieved significant progress in child health indicators. The main reason behind this progress was found to be diligent supervision, review meetings, instructional circular and innovative programs to reach the people dwelling in slums [59]. Furthermore, the concentration of both allopathic doctors and nurses is relatively higher than the population concentration of Maharashtra. In contrast, the density of the allopathic doctors in the North-Eastern states is the lowest among the Indian states [60]. Mizoram (EI = 0.36) witnessed the highest level of inequality, closely followed by other North-Eastern states. A study conducted in the North-Eastern region discerned that the children suffering from febrile illness sought care from either traditional healers or relied on self-medication. It mainly occurred due to the transportation hurdles associated with the remoteness of the locality [16]. Although there was some improvement in the rural health infrastructure in the North-Eastern states in the post-NRHM phase, the condition of the region is still dreadful, as these states suffer from a severe shortage of specialists and trained health personnel [61]. This might be why the present study also found that the interspace between the prevalence and treatment of illness within each wealth quintile was higher in the North-Eastern states than in the other states.

## Limitations

The data used in this study does not have a broader scope for the need-based factors of child health. Other biological indicators of child health can be used to get a better picture of the inequity analysis. The study may suffer from recall bias, as it depends on the precision of the respondent’s memory, who provided information about the child’s health in the last 5 years. The study could not capture the micro-inequities that always prevail in the utilization of health care among the vulnerable sections of the population. These issues can be taken into consideration for further studies.

## Conclusion

The findings of this study can be used to formulate or reshape child health care policies. The study reveals that the horizontal inequity in the utilization of medical treatment for children is pro-rich in India. The continuum of maternal health care was found to be a significant contributor to the pro-rich inequity. Hence, it is imperative to strengthen the pathway of the continuum of maternal health care, and efforts have been made in this aspect. However, the government can make further efforts to integrate the continuum of maternal health and child health care services into one seamless chain of care. These initiatives may ameliorate inequalities and improve the utilization of maternal and child health care services.

The provision of services does not ensure that women will use them; they first need to be aware of the available services and then perceive them as beneficial for their children. The role of media exposure is crucial in this context. The media can be used as a powerful tool in pursuing mothers to utilize health care services. Furthermore, the mother’s education always has a critical role to play. Though increasing literacy is always the desirable goal, the outcome will be visible only in the long run. Therefore, the policy community could focus on spreading health education through various means to increase awareness among mothers about the necessity of seeking care for childhood diseases. Besides, affordability is one of the primary barriers to access child health care services. This barrier can be overcome through public financial assistance/transfer schemes. In addition, reaching out to rural places with adequate health personnel could accelerate the pace of utilization. The Government of India has launched the Ayushman Bharat scheme to remove the poor’s financial hardships related to health care services. It is expected that the intensive implementation of this program for child health care services might be able to pave the way for achieving remarkable progress in child health. The present study could be used as a stepping stone towards understanding the socio-economic components of inequity in medical treatment utilization for common illnesses among children in India or any other developing country with similar contextual factors.

## Data Availability

The dataset used for the current study is available on the DHS Program website, https://dhsprogram.com/data/available-datasets.cfm.The data is available for public access and can be accessed for free after a simple registration process and permission approved by the Demographic and Health Survey (DHS) Program.

## Appendix 1

### State groups

The states were grouped adhering to the National Rural Health Mission criterion of **high focus** and **non-high focus states**. *Non-high focus states* are Andhra Pradesh, Goa, Gujarat, Haryana, Karnataka, Kerala, Maharashtra, Punjab, Tamil Nadu, West Bengal, and Telangana. Further, we have divided high focus states into two groups- *major high focus states* (Bihar, Chhattisgarh, Himachal Pradesh, Jammu & Kashmir, Jharkhand, Madhya Pradesh, Odisha, Rajasthan, Uttar Pradesh, and Uttarakhand) and *high focus North-East states* (Arunachal Pradesh, Assam, Manipur, Meghalaya, Mizoram, Nagaland, Sikkim, Tripura) considering the geographical location (mountainous area) and budgetary allocation (special state provision) to North-Eastern region.

## Abbreviations

ARI: Acute Respiratory Infection
CI: Concentration Index
CC: Concentration curve
CCHI: Common childhood Illnesses
CoC: Continuum of maternal care
BASEP: Basic amenities and socioeconomic profile
EI: Erreygers Corrected Concentration Index
HII: Horizontal Inequity Index

## Footnotes

1 The Government of India launched NRHM in 2005. In 2013, the mission extended to urban areas as the National Urban Health Mission (NUHM). Both the missions are now working as two sub-missions of the National Health Mission (NHM). The NHM envisages achieving equitable, affordable and quality health care services for all.
